# Intraoperative Tips for Hand Surgery: A Focus on Reducing Postoperative Scarring and Tendon Rupture

**DOI:** 10.7759/cureus.3274

**Published:** 2018-09-10

**Authors:** Andrew Sephien, Katherine Peters, Christopher Shoji, Francisco A Schwartz-Fernandes

**Affiliations:** 1 Orthopaedics and Sports Medicine, University of South Florida Morsani College of Medicine, Tampa, USA

**Keywords:** tendon rupture, intraosseous tunneling, plate deburring, wound healing, scar minimization, surgical techniques

## Abstract

This article is a collection of intraoperative techniques performed by a single hand surgeon with literature support for these techniques for the purpose of circumventing potential limitations intraoperatively. These techniques include the use of a Beaver Blade handle (Beaver, Beaver-Visitec, Waltham, MA, US) to be used as a rasp to smooth intraosseous tunnels during tendon transfers, a Stryker (Stryker, Stryker Corporation, Kalamazoo, MI, US) or Synthes (Deputy Synthes, Johnson & Johnson, West Palm Beach, FL, US) drill as a motorized file for plate deburring, and Insorb forceps (Insorb, Incisive Surgical, Plymouth, MN, US) for skin closure. These tips serve as methods to minimize scarring and circumvent unfortunate obstacles, such as tendon rupture, and the consequential weakened repair that can occur post-operatively. These have not been previously reported in the literature but have been performed by the senior author with no resulting complications. Additionally, the common availability of the equipment allows for a potential economic benefit.

## Introduction

This article is a collection of the author's innovative techniques, utilizing commonplace equipment in place of expensive and limited-use tools, for hand surgery procedures with the support of literature on the use of these techniques for the purpose of saving time and circumventing potential limitations. This paper aims to share the creativity and insights of a single hand surgeon, who is the senior author, so that fellow surgeons may use his experiences to become more productive in the surgical setting. These technical shortcuts have not been previously reported in the literature but have been performed by the senior author with no resulting complications and can be performed using commonly available tools, thus providing economic efficiency, particularly in settings where the “standard” tools used may not be available. These techniques include the use of a Beaver Blade handle (Beaver, Beaver-Visitec, Waltham, MA, US) to be used as a rasp to smooth intraosseous tunnels during tendon transfers, the use of a Stryker (Stryker, Stryker Corporation, Kalamazoo, MI, US) or Synthes (Deputy Synthes, Johnson & Johnson, West Palm Beach, FL, US) drill as a motorized file for plate deburring, and Insorb (Insorb, Incisive Surgical, Plymouth, MN, US) forceps for skin closure. These tips serve as methods to minimize scarring and circumvent unfortunate complications, such as tendon rupture, and the consequential weakened repair that can occur to patients postoperatively. We present three different techniques using the existing literature to support the utility of these techniques.

## Technical report

The use of a Beaver Blade handle to smooth intraosseous tunnels for tendon transfers

**Figure 1:**
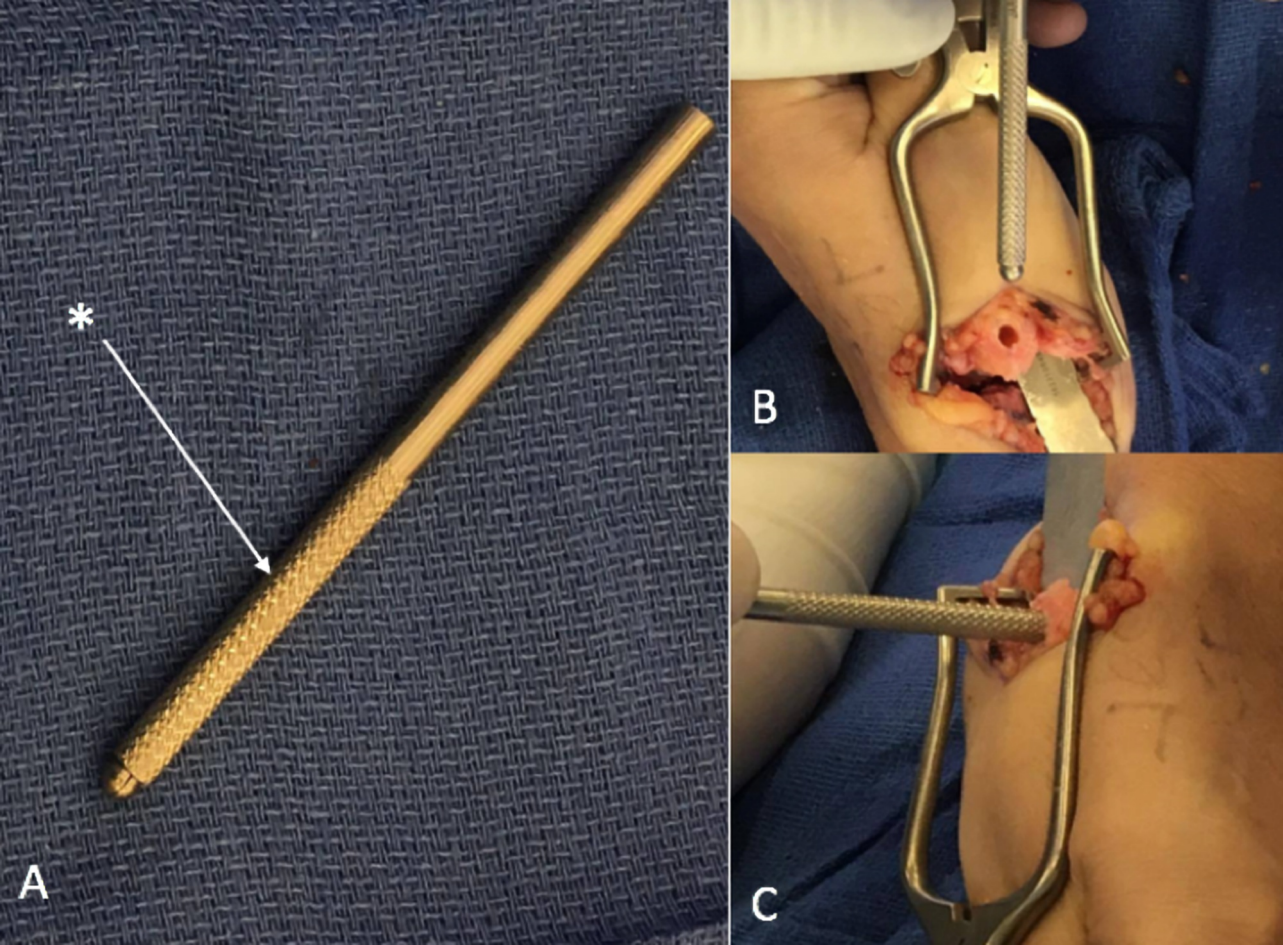
Beaver Blade: the use of the knurled portion (*) of a Beaver Blade (A) is used to enlarge an intraosseous tunnel (B and C) and deburr any potential edges. Beaver Blade (Beaver, Beaver-Visitec, Waltham, MA, US)

The use of a rasp in orthopedic surgery is an important step in shaping, contouring, trimming, and forming bone surfaces and removing bone irregularities in the area of interest [1]. Additionally, the creation of intraosseous tunnels is commonly used in hand surgeries and has become an alternative technique in other surgical operations [2-3]. The exploration of an intraosseous technique in other surgeries was prompted due to the unfortunate complications of anchor repair, such as the difficulty in the re-tear of a repair due to the attachment of previous anchors and the cost of anchors [2]. While there are many types and sizes of burring instruments, it is important to realize that not all instruments may be available, and it is sometimes necessary to adaptively use the tools available to continue with the procedure. In our modifications, we smooth out the intraosseous tunnel with the knurled portion of a Beaver Blade handle as a rasp to deburr and remove any potential edges inside the tunnel while, at the same time, enlarging the tunnel without the need of using a drill bit (Figure 1). In the event that the Beaver Blade handle is not available, an alternative is to use skin hooks or any other instrument handle that can behave as a rasp to smoothen out the tunnel.

The use of a Stryker or Synthes drill as a motorized file for plate deburring

**Figure 2:**
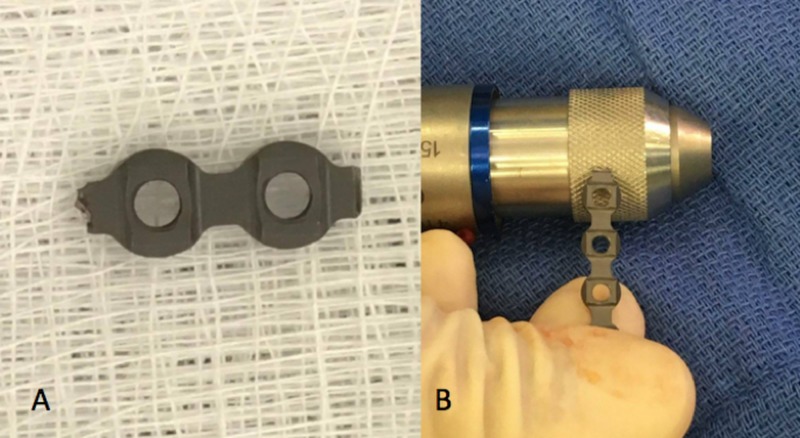
Plate deburring. Prominent edges demonstrated on plates (A) can be deburred through the use of a Synthes/Striker drill (B). Stryker, Stryker Corporation, Kalamazoo, MI, US; Synthes Deputy Synthes, Johnson & Johnson, West Palm Beach, FL, US

The rupture or irritation of tendons after fracture fixation is a known complication and can easily be prevented by removing all the unnecessary sharp edges from metallic implants to protect the soft tissues [4-5]. Recent literature has shown that implant prominence is also a significant factor in tendon rupture, with some reports citing it as a cause of flexor tendon rupture [4,6]. The force and pressure on a tendon can increase significantly with even a small shift in plate placement [7]. This technique involves the use of a Stryker or Synthes drill as a motorized file to circumvent the risk of tendon rupture (Figure 2). The drill allows the modified plate to be deburred at “high speed,” providing precision, time efficacy, and smoother edges as compared to manual filing. It is worthy of note that it is unlikely that this process will damage the drill and should not be of concern to the user.

The use of an Insorb forceps for skin closure to optimize wound healing and minimize scarring

**Figure 3:**
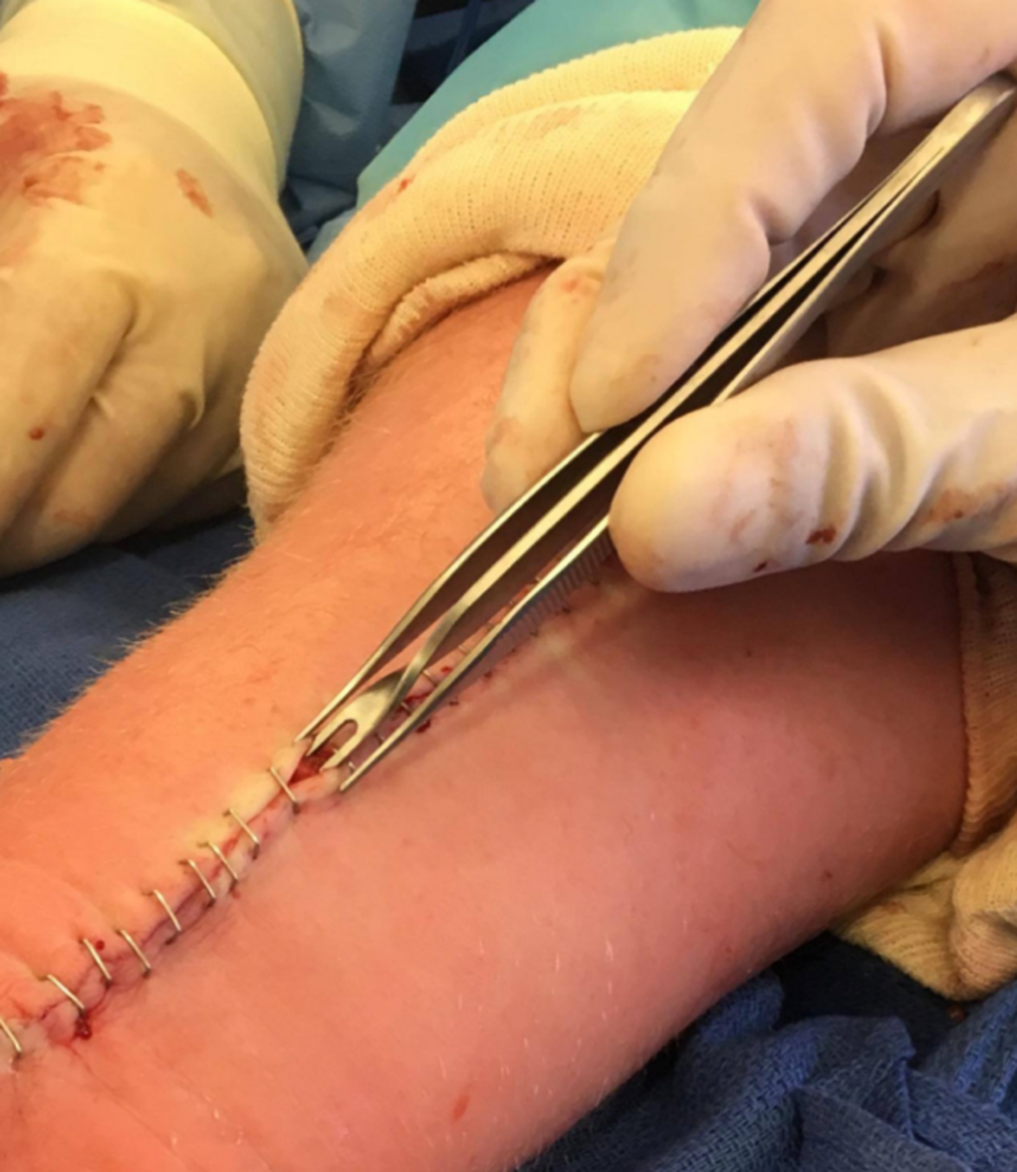
Insorb forceps: the use of an Insorb forceps (shown above) allows for wound eversion during stapling. Insorb, Incisive Surgical, Plymouth, MN, US

The concept of wound healing is a dynamic, physiological process consisting of biological mechanisms in both inflammation and tissue regrowth [8-9]. Proper wound closure following surgery is essential for preventing complications and minimizing scaring [8,10]. Proper healing can be achieved by aligning the wound tissue in such a manner that opposing skin is matched with its proper anatomy and vasculature. Optimal wound healing can be further stimulated through an eversion of the wound tissue rather than an inversion due to the natural inversion of skin wounds upon healing [11]. To aid eversion while using metal staples, the senior author uses the Insorb double arm forceps, originally designed for a subcutaneous absorbable skin stapler (Figure 3).

## Discussion

Using a Beaver Blade handle to smooth intraosseous tunnels for tendon transfers

In basal joint arthritis, the creation of intraosseous tunnels is standard when weaving the flexor carpi radialis (FCR) through the first metacarpal. Normally, ligament reconstruction is performed by guiding the FCR through a channel at the first metacarpal, and the remainder of the tendon is coiled and inserted to create a spacer between the metacarpal and the carpal bones of the wrist [3]. With the creation of a spacer, there is a potential for the creation of a bone prominence in the tunnel, which allows for the opportunity to snag and strip the tendon, ultimately resulting in a weakened tendon, leading to rupture and weak repair. In an effort to reduce this significant complication, this technique has been performed by the senior author in 35 cases over the past year and a half, with no complication of tendon rupture.

Using a Stryker or Synthes drill as a motorized file for plate deburring

The risk of rupture can be reduced through diligence in plate fixation [7]. This may include altering the plate length to reduce plate prominence in surgery [12]. In altering plate length in hand surgery, such as in the use of interphalangeal plates, deburring adjustments are necessary post the alteration to ensure that the plate size reduction does not unexpectedly cause tendon rupture. The use of a Stryker or Synthes drill as a motorized file for plate deburring has been performed by the senior author in a total of 10 cases, in an effort to reduce tendon rupture. In all 10 cases, there were no complications of tendon rupture. This technique has not been reported in the literature, and we believe it can minimize the rupture risk by reducing the force and pressure on the tendon, therefore leading to better patient outcomes.

Using an Insorb forceps for skin closure to optimize wound healing and minimize scarring

Normal healing mechanisms promote wound contraction and eversion [13]. However, an inaccurate matching of skin tissue can prevent proper tissue homeostasis from occurring [8,11]. If caution is not taken and wound closure is concluded with inaccurate skin matching, resulting in inverted skin edges, the risk of unsightly or prominent scar formation increases significantly [13]. This emphasizes the importance of eversion in wound healing to minimize potential scarring and optimize healing. The Insorb double arm forceps is commonly used by the senior author to promote eversion. The forceps' design allows for the opposing edges of the wound to be everted during stapling, which promotes optimal wound healing due to the increased likelihood of proper skin layer alignment and compensation for the natural eversion [9].

## Conclusions

In efforts to reduce the complications of tendon rupture and the resulting weakness of tendon repairs or tendon transfers and increase cosmetically acceptable wound closure, this article presents three intraoperative techniques developed by the author over several years of practice. These techniques include the use of a Beaver Blade handle to be used as a rasp to smooth intraosseous tunnels during tendon transfers, the use of a Stryker or Synthes drill as a motorized file for plate deburring, and Insorb forceps for skin closure. These techniques have not been previously reported in the literature but have been developed over several years in practice by the senior author, simplifying the equipment needed to perform common hand surgery procedures while reducing complications.
